# Detection of *Theileria orientalis* Genotypes from Cattle in Kyrgyzstan

**DOI:** 10.3390/pathogens11101185

**Published:** 2022-10-14

**Authors:** Sezayi Ozubek, Mehmet Can Ulucesme, Veli Yılgor Cirak, Munir Aktas

**Affiliations:** 1 Department of Parasitology, Faculty of Veterinary Medicine, University of Firat, Elazig 23119, Turkiye; 2 Department of Parasitology, Faculty of Veterinary Medicine, Bursa Uludag University, Bursa 23119, Turkiye

**Keywords:** *Theileria* *orientalis*, genotype, cattle, PCR

## Abstract

The ikeda and chitose genotypes of *Theileria orientalis*, which for many years were thought to be benign, cause a disease that results in significant economic losses in the cattle industry. This study was carried out in order to determine the genotypes of *T. orientalis* in cattle in Kyrgyzstan, and 149 archived DNA samples known to be *T. orientalis* were analyzed by the PCR amplification of the major piroplasm surface protein (MPSP) gene region. Single-Strand Conformation Polymorphism (SSCP) analysis was performed to uncover the nucleotide changes in the archived DNA samples, and 15 samples showing different band profiles were subjected to sequence analysis. As a result of the sequence analysis, it was seen that the samples belonged to the buffeli and chitose A genotypes. In order to identify mixed genotypes, PCR was performed using primers specific for these genotypes, and buffeli (type 3), chitose (type 1) and buffeli+chitose were found to be positive in 26.2%, 2% and 71.8% of samples, respectively. As a result of this study, we showed the presence of buffeli (type 3) and chitose (type 1) genotypes of *T. orientalis* in cattle in Kyrgyzstan. Comprehensive epidemiological studies are needed to understand the clinical infections caused by the pathogenic chitose A and to determine the geographical distribution and different genotypes of *T. orientalis*.

## 1. Introduction

Bovine theileriosis, caused by *Theileria* species (Apicomplexa: Piroplasmida; Theileriidae), is an important tick-borne disease of cattle in tropical and subtropical regions of the world and causes serious economic losses [1,2]. It is known that *Theileria parva* and *Theileria annulata*, which are also known as transforming species, are highly pathogenic species for cattle. Other species of *Theileria* that infect cattle, *Theileria mutans*, *Theileria taurotragi*, and members of the *Theileria orientalis* complex, frequently cause benign infections [3,4,5]. It has been observed that *Theileria orientalis*, long thought to be benign, has different genotypes, some of which cause clinical cases and adversely affect the cattle industry [6]. According to sequence variations in the *major piroplasm surface protein* (*MPSP*) gene, 11 genotypes, type 1 (chitose), type 2 (ikeda), type 3 (buffeli), type 4–8 and N1-N3, have been reported in various region in the world. Of these genotypes, ikeda and chitose cause clinical cases of oriental theileriosis in cattle. The transmission of *T. orientalis* occurs through the feeding of infected ticks of the *Haemaphysalis* genus [7,8]. It has also been reported that *T. orientalis* can be spread mechanically among cattle via blood-sucking flies and arthropods. [9]. Recently, it has been shown that sheep can be effective in spreading *T. orientalis*; healthy sheep can be infected with the pathogenic genotype ikeda, and ticks can become infected by feeding on sheep [10].

In a molecular survey we conducted in 2019, we reported that *T. orientalis* is the most common blood parasite (32.8%; CI 28.5–37.3) in cattle in Kyrgyzstan [11]. In this study, we aimed to determine which genotypes of *T. orientalis* are present in cattle in Kyrgyzstan.

## 2. Materials and Methods

**Figure 1:**
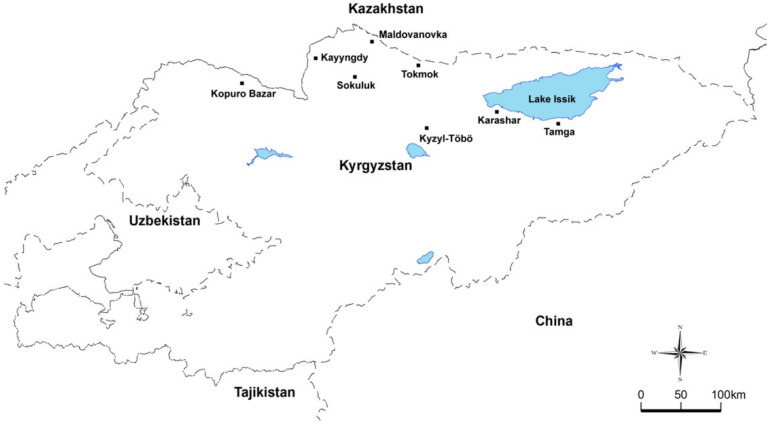
Map of Kyrgyzstan and sampling regions.

Archived DNA samples used in this study and detailed information from the investigated provinces has been documented previously [11]. Briefly, this study was carried out between December 2012 and June 2013 on 454 cattle in eight provinces (Karaşar, Kayyngdy, Kızıl-Töbö, Kopuro Bazar, Moldovanovka, Sokuluk, Tamga, and Tokmok) located around the Chu valley and Issyk Kul Lake in Kyrgyzstan (Figure 1). All DNA samples were tested using the reverse line blot assay for bovine *Theileria* and *Babesia* species to determine the frequency of piroplasm distribution, and 149 DNA samples identified as containing *T. orientalis* [11] were used for genotype analysis.

### PCR Amplification, Single-Strand Conformation Polymorphism (SSCP) and Phylogenetic Analysis

To investigate the genetic diversity of *T. orientalis*, the *Major Piroplasm Surface Protein* (*MPSP*) gene was amplified by nested PCR. Briefly, the primers MPSP-F/MPSP-R [12] were used for the initial amplification of the *MPSP* gene in *T. orientalis*. Nested amplification was performed using the primers MPSPAJ-F/MPSP-AJ-R1 [13]. Ten microliters of the PCR products were used on 1.6% agarose gel for visualization and the remaining products were stored at 4 °C until use in SSCP. Single-Strand Conformation Polymorphism was performed to see possible sequence changes in the *MPSP* gene, and samples which had different band profiles were sent for sequence analysis. After sequence analysis, phylogenetic analysis was performed using the MEGAX program [14] and genotypes were determined accordingly. Genotype-specific PCR was then performed to determine whether infections of mixed genotype were present, using primers TSB-TSR (buffeli) [15], TSC-TSR (chitose) [16] and TSI-TSR (ikeda) [17] (Table 1). *Theileria orientalis* genomic DNA, previously detected by PCR and DNA sequencing (GenBank accession number MK415835), were used as positive controls in the PCR.

## 3. Results

**Figure 2:**
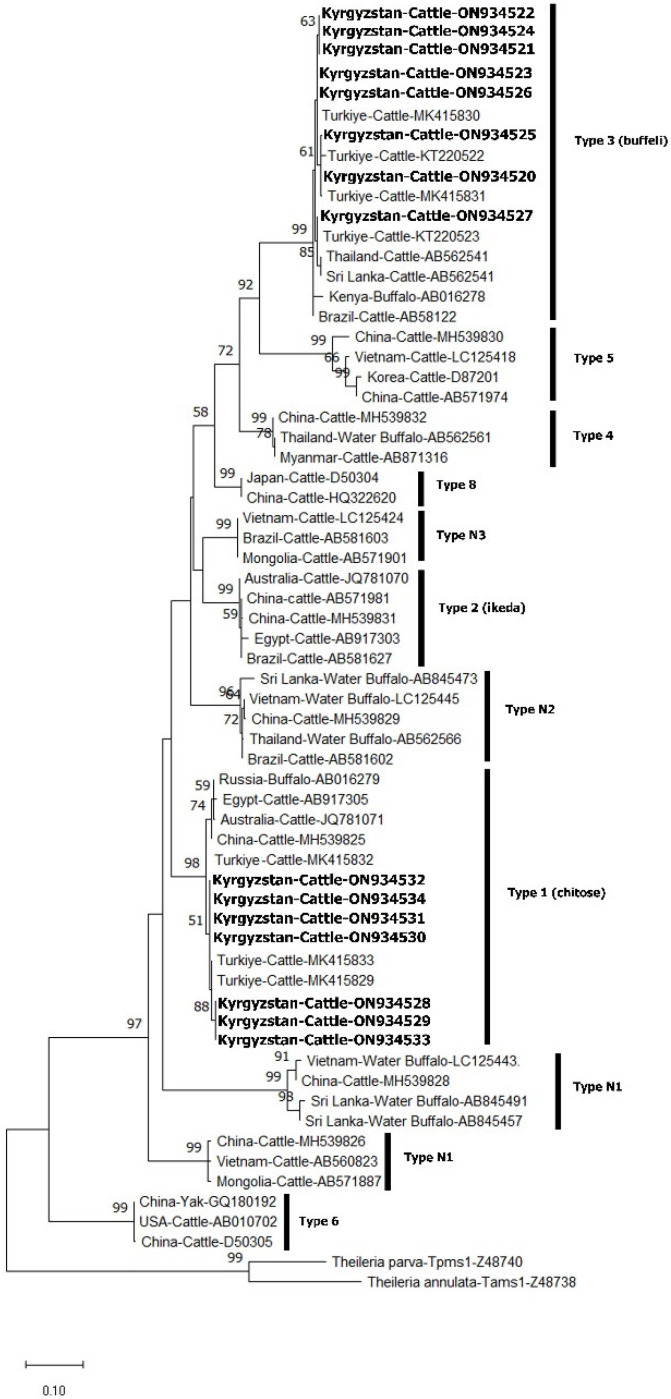
Phylogenetic analysis by maximum likelihood using *T. orientalis MPSP* gene sequences. The tree constructed using the Tamura 3-parameter model shows the phylogenetic relationship of the *T. orientalis* genotypes identified in this study (in bold) with other genotypes obtained from GenBank. The analysis includes 64 nucleotide sequences, and the percentage of the replica tree (1000 copies) in which related taxa clustered together in the bootstrap test are shown next to the branches.

**Figure 3:**
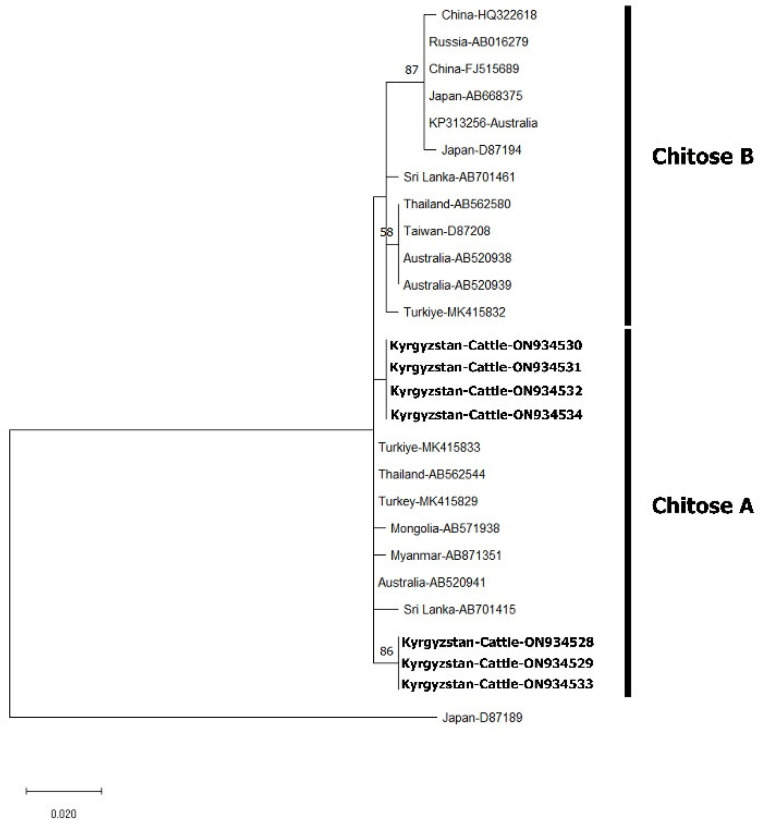
Phylogenetic analysis by maximum likelihood using chitose genotypes *MPSP* gene sequences. The tree constructed using the Kimura 2- parameter model shows the phylogenetic relationship of the *T. orientalis* genotypes identified in this study (in bold) with other genotypes obtained from GenBank. The analysis includes 27 nucleotide sequences, and the percentage of the replica tree (1000 copies) in which related taxa clustered together in the bootstrap test are shown next to the branches.

All 149 *T. orientalis* samples were amplified by nested PCR for the *MPSP* gene. The amplicons obtained from these samples were used in SSCP analysis and 15 samples with different band profiles were sent for sequence analysis (Supplementary Figure S1). The nucleotide sequences have been registered in GenBank under the accession numbers ON934520- ON934534. A maximum likelihood phylogenetic analysis based on the Tamura 3-parameter model [18] was created using the sequences obtained as a result of the sequence analysis and the sequences of 11 *T. orientalis* genotypes obtained from GenBank (Figure 2). As a result of phylogenetic analysis, it was seen that the samples belonged to buffeli and chitose genotypes. Furthermore, phylogenetic analysis was performed using the Kimura 2-parameter model [19] to determine chitose genotypes and it was seen that all samples belonged to the chitose A genotype (Figure 3). As a result of genotype-specific PCR performed to determine mixed genotypes, buffeli, chitose and buffeli+chitose were found to be 26.2%, 2% and 71.8%, respectively (Table 2).

## 4. Discussion

Livestock, which is an important part of the economy of Kyrgyzstan, is at the forefront of the livelihoods for the majority of the population [20,21]. Tick-borne diseases (TBDs) have a significant economic impact on livestock worldwide. Epidemiological studies of tick-borne infections are crucial to identify tick–host–pathogen interactions; characterize pathogen transmission, occurrence, and pathogenesis; and identify new checkpoints for the control of both vectors and pathogens [22,23]. However, there are very few studies on TBDs on cattle in Kyrgyzstan. Aktas et al. [11] have reported *Babesia major*, *T. annulata* and *T. orientalis* in cattle in Kyrgyzstan using molecular methods (PCR-RLB). Altay et al. [24] have revealed the presence of *Anaplasma capra*, *Anaplasma centrale*, and *Anaplasma phagocytophilum* like-1 from cattle in Kyrgyzstan. *Anaplasma capra* and *A. phagocytophilum* are also known to cause serious infections in humans. Although there is limited information on TBD infections in farm animals in Kyrgyzstan, more comprehensive studies have been observed in countries that border Kyrgyzstan, especially China. In China, *T. orientalis* is the most common *Theileria* species, and all genotypes of *T. orientalis* have been identified from field-collected blood samples or ticks [25]. *Anaplasma marginale* and *Babesia bigemina* have been reported serologically in cattle in Tajikistan [26]. Live vaccines against infections caused by *T. annulata* and *B. bigemina* have been developed and applied in Uzbekistan [27,28]. There is no information about *T. orientalis* genotypes in Kazakhstan, Tajisiktan, and Uzbekistan located on the border of Kyrgyzstan.

*Theileria orientalis*, which consists of multiple genotypes and is recommended to be classified as a single species complex, can cause significant infections in cattle. Ikeda and chitose, known as virulent genotypes that cause pathogenic infections, have been reported in several countries, including Australia [29], New Zealand [30] and, more recently, the USA [31]. The molecular prevalence of *T. orientalis* in cattle in Kyrgyzstan was reported as 32.8% [11]. In this study, these samples have been shown to belong to buffeli and chitose genotypes. The buffeli genotype is largely considered a benign, non-pathogenic component of the *T. orientalis* complex [2,32]. Previous infection with the buffeli genotype has also been reported to be protective against the pathogenic ikeda genotype of *T. orientalis* [33]. The chitose genotype that causes clinical infections of *T. orientalis* is divided into two groups, chitose A and chitose B. In addition, Chitose A genotype has been found to be more pathogenic than chitose B, although there is little information [34]. In this study, all the samples defined as the chitose genotype were determined to belong to the chitose A genotype according to the phylogenetic analysis. Only two genotypes of T. orientalis were identified in this study; however, further investigation is warranted, including the sampling of cattle in the southeastern region, especially close to the border of China, in order to determine whether different genotypes are present in these regions.

The economic loss due to the pathogenic genotype of the *T. orientalis* amounts to millions of dollars annually. Integrated Parasite Management (IPM) is applied to reduce these economic losses [35]. In addition, occult carriers, such as sheep, have been found to be important in the spread of *T. orientalis* as a source of infection for naive ticks [10]. It has also been reported that the buffeli genotype may be protective against pathogenic genotypes. As reported in this study, although the buffeli genotype is common in Kyrgyzstan, it should be kept in mind that there may be clinical cases originating from imported cases and the disease may spread more rapidly with the presence of the *Haemaphysalis longircornis* ticks [36]. Although a few tick species belonging to the genus *Haemaphysalis* (*Haemaphysalis punctate*, *Haemaphysalis erinacei*) have been reported in Kyrgyzstan [37], there are no data on *H. longicornis*, the main vector of *T. orientalis*.

## 5. Conclusions

In conclusion, two genotypes of *T. orientalis* in cattle have been reported in this study. Of these, the chitose A genotype is pathogenic and economically important for the cattle industry. Although the existence of ticks that can spread TBDs agents in Kyrgyzstan has been reported [37], there are no data on clinical infections caused by TBDs. In the future, detailed information about the geographical distribution, vector compatibility, different genotypes, and the clinical pathologies of *T. orientalis* can be obtained with epidemiological studies.

## Figures and Tables

**Table 1:** The primers used in the study.

Primer Name	Primer Sequence 5′-3′	PCR Condition	References
MPSP-F	CTTTGCCTAGGATACTTCCT	95 °C, 3 min; 95 °C, 30 s; 58 °C, 30 s; and 72 °C, 30 s (35 cycles); final extension of 72 °C, 5 min.	[12]
MPSP-R	ACGGCAAGTGGTGAGAACT		
MPSP-AJ-F	TTCACTCCAACAGTCGCCCACA	95 °C, 3 min; 95 °C, 30 s; 60 °C, 30 s; and 72 °C, 30 s (35 cycles); final extension of 72 °C, 5 min.	[13]
MPSP-AJ-R1	ACGTAAACTTTGACTGCGGTG		
TSB	CACCTTCCTCATCGTCTCTGCAACT	95 °C, 3 min; 95 °C, 30 s; 55 °C, 30 s; and 72 °C, 30 s (35 cycles); final extension of 72 °C, 5 min.	[15]
TSR	CACCTGCTCTGCAACCGCAGAG		
TSC	CACCTTCCTCATCGTCTCTGCAACT	95 °C, 3 min; 95 °C, 30 s; 55 °C, 30 s; and 72 °C, 30 s (35 cycles); final extension of 72 °C, 5 min.	[16]
TSR	CACCTGCTCTGCAACCGCAGAG		
TSI	CACCATCGTCTGCTACCGCCGC	95 °C, 3 min; 95 °C, 30 s; 55 °C, 30 s; and 72 °C, 30 s (35 cycles); final extension of 72 °C, 5 min.	[17]
TSR	CACCTGCTCTGCAACCGCAGAG		

**Table 2:** In this study, *T. orientalis* genotypes determined in different provinces of Kyrgyzstan.

	Number of *T. orientalis* Genotypes				
Province	No. of Positive Samples	Type 1 (Chitose)	Type2 (Ikeda)	Type 3 (Buffeli)	Type 1 + Type 3
Tokmok	13	1	-	2	10
Sokuluk	22	1	-	7	14
Karashar	3	-	-	-	3
Kyzyl-Töbö	3	-	-	1	2
Kopuro Bazar	19	-	-	5	14
Tamga	2	-	-	-	2
Kayyngdy	27	-	-	8	19
Maldovanovka	60	1	-	16	43
Total	149	3 (2%)	-	39 (26.2%)	107 (71.8%)

## Data Availability

Data available in a publicly accessible repository.

## Supplementary Materials

The following supporting information can be downloaded at: https://www.mdpi.com/article/10.3390/pathogens11101185/s1, Figure S1: Representative single-stranded conformation polymorphism gel showing 15 different profiles (P1–P7).

## References

[1] Aktas M., Altay K., Dumanli N. (2006). A molecular survey of bovine *Theileria* parasites among apparently healthy cattle and with a note on the distribution of ticks in eastern Turkey. Vet. Parasitol..

[2] Sivakumar T., Hayashida K., Sugimoto C., Yokoyama N. (2014). Evolution and genetic diversity of *Theileria*. Infect. Genet. Evol..

[3] Uilenberg G., Perie N., Spanjer A., Franssen F. (1985). *Theileria orientalis*, a cosmopolitan blood parasite of cattle: Demonstration of the schizont stage. Vet. Sci. Res. J..

[4] Uilenberg G. (2011). *Theileria sergenti*. Vet. Parasitol..

[5] Woods K., Perry C., Brühlmann F., Olias P. (2021). *Theileria*’s strategies and effector mechanisms for host cell transformation: From invasion to immortalization. Front. Cell Dev. Biol..

[6] Eamens G., Bailey G., Gonsalves J., Jenkins C. (2013). Distribution and temporal prevalence of *Theileria orientalis* major piroplasm surface protein types in eastern Australian cattle herds. Aust. Vet. J..

[7] Riek R. (1982). Epidemiology and transmission of *Theileria* sp. of cattle in Australia. Aust. Vet. J..

[8] Stewart N., De Vos A., Shiels I., McGregor W. (1987). The experimental transmission of *Theileria buffeli* of cattle in Australia by *Haemaphysalis humerosa*. Aust. Vet. J..

[9] Lakew B.T., Kheravii S.K., Wu S.-B., Eastwood S., Andrew N.R., Nicholas A.H., Walkden-Brown S.W. (2021). Detection and distribution of haematophagous flies and lice on cattle farms and potential role in the transmission of *Theileria orientalis*. Vet. Parasitol..

[10] Lawrence K., Gedye K., Hickson R., Wang B., Carvalho L., Zhao Y., Pomroy W. (2021). The role of sheep (*Ovis aries*) in maintaining *Theileria orientalis* Ikeda type infection. Vet. Parasitol..

[11] Aktas M., Kısadere I., Ozubek S., Cihan H., Salıkov R., Cirak V.Y. (2019). First molecular survey of piroplasm species in cattle from Kyrgyzstan. Parasitol. Res..

[12] Ota N., Mizuno D., Kuboki N., Igarashi I., Nakamura Y., Yamashina H., Hanzaike T., Fujii K., Onoe S., Hata H. (2009). Epidemiological survey of *Theileria orientalis* infection in grazing cattle in the eastern part of Hokkaido, Japan. J. Vet. Med. Sci..

[13] Cufos N., Jabbar A., de Carvalho L.M., Gasser R.B. (2012). Mutation scanning-based analysis of *Theileria orientalis* populations in cattle following an outbreak. Electrophoresis.

[14] Kumar S., Stecher G., Li M., Knyaz C., Tamura K. (2018). MEGA X: Molecular evolutionary genetics analysis across computing platforms. Mol. Biol. Evol..

[15] Kubota S., Sugimoto C., Kakuda T., Onuma M. (1996). Analysis of immunodominant piroplasm surface antigen alleles in mixed populations of *Theileria sergenti* and *T. buffeli*. Int. J. Parasitol..

[16] Matsuba T., Kubota H., Tanaka M., Hattori M., Murata M., Sugimoto C., Onuma M. (1993). Analysis of mixed parasite populations of *Theileria sergenti* using cDNA probes encoding a major piroplasm surface protein. Parasitology.

[17] Kawazu S.-i., Kamio T., Kakuda T., Terada Y., Sugimoto C., Fujisaki K. (1999). Phylogenetic relationships of the benign *Theileria* species in cattle and Asian buffalo based on the major piroplasm surface protein (p33/34) gene sequences. Int. J. Parasitol..

[18] Tamura K. (1992). Estimation of the number of nucleotide substitutions when there are strong transition-transversion and G+ C-content biases. Mol. Biol. Evol..

[19] Kimura M. (1980). A simple method for estimating evolutionary rates of base substitutions through comparative studies of nucleotide sequences. J. Mol. Evol..

[20] Zhumanova M., Maharjan K.L. (2012). Trends in livestock population and composition through derived productivity in Kyrgyzstan: A case study in Ala-Buka District. J. Int. Dev. Coop..

[21] Karadag H. (2019). Bağımsızlık Sonrası Kırgızıstan’da Hayvancılıkta Gelişmeler. YYUSBED.

[22] Wikel S.K. (2018). Ticks and tick-borne infections: Complex ecology, agents, and host interactions. Vet. Sci..

[23] Schnittger L., Ganzinelli S., Bhoora R., Omondi D., Nijhof A.M., Florin-Christensen M. (2022). The Piroplasmida *Babesia*, *Cytauxzoon*, and *Theileria* in farm and companion animals: Species compilation, molecular phylogeny, and evolutionary insights. Parasitol. Res..

[24] Altay K., Erol U., Sahin O.F., Aytmirzakizi A. (2022). First molecular detection of *Anaplasma* species in cattle from Kyrgyzstan; molecular identification of human pathogenic novel genotype *Anaplasma capra* and *Anaplasma phagocytophilum* related strain. Ticks Tick-Borne Dis..

[25] Liu J., Li Z., Liu A., Wang J., Guan G., Yin H., Luo J. (2022). Identification and isolation of pathogenic *Theileria orientalis* Ikeda genotype from confined dairy cattle, in Hebei, China. Parasitol. Res..

[26] Gralén B. (2009). Tick-Borne Diseases in Tajikistan.

[27] Shkap V., Rasulov I., Abdurasulov S., Fish L., Leibovitz B., Krigel Y., Molad T., Mazuz M., Savitsky I. (2007). *Babesia bigemina*: Attenuation of an Uzbek isolate for immunization of cattle with live calf-or culture-derived parasites. Vet. Parasitol..

[28] Rasulov I., Fish L., Shkap V. (2008). Vaccination of cattle against tropical theileriosis in Uzbekistan using autochthonous live vaccine. Vaccine.

[29] Kamau J., de Vos A.J., Playford M., Salim B., Kinyanjui P., Sugimoto C. (2011). Emergence of new types of *Theileria orientalis* in Australian cattle and possible cause of theileriosis outbreaks. Parasites Vectors.

[30] McFadden A., Rawdon T., Meyer J., Makin J., Morley C., Clough R., Tham K., Müllner P., Geysen D. (2011). An outbreak of haemolytic anaemia associated with infection of *Theileria orientalis* in naive cattle. N. Z. Vet. J..

[31] Oakes V.J., Yabsley M.J., Schwartz D., LeRoith T., Bissett C., Broaddus C., Schlater J.L., Todd S.M., Boes K.M., Brookhart M. (2019). *Theileria orientalis* Ikeda genotype in cattle, Virginia, USA. Emerg. Infect. Dis..

[32] Watts J., Playford M., Hickey K. (2016). *Theileria orientalis*: A review. N. Z. Vet. J..

[33] Emery D., de Burgh S., Dinh T.H.H.H., Rolls P., Carter P. (2021). Merozoites of *Theileria orientalis* buffeli reduce the parasitaemia of *T. orientalis* ikeda following tick challenge. Vet. Parasitol..

[34] Jenkins C., Micallef M., Alex S., Collins D., Djordjevic S., Bogema D. (2015). Temporal dynamics and subpopulation analysis of *Theileria orientalis* genotypes in cattle. Infect. Genet. Evol..

[35] Emery D.L. (2021). Approaches to Integrated Parasite Management (IPM) for *Theileria orientalis* with an Emphasis on Immunity. Pathogens.

[36] Lawrence K., Gedye K., McFadden A., Pulford D., Heath A., Pomroy W. (2021). Re-view of the New Zealand *Theileria orientalis* Ikeda Type Epidemic and Epidemiologi-cal Research since 2012. Pathogens.

[37] Fedorova S.Z. (2005). Ixodidae ticks in Bishkek. Meditsinskaia Parazitol. Parazit. Bolezn..

