# Supplementary material for: Detection of Theileria orientalis Genotypes from Cattle in Kyrgyzstan
Source: Pathogens. 2022 Oct 14;11(10):1185. doi: 10.3390/pathogens11101185 (PMC9606894; doi:10.3390/pathogens11101185)
Supplement: Supplementary file 1 [file pathogens-11-01185-s001.zip › pathogens-1945186-supplementary.pdf]

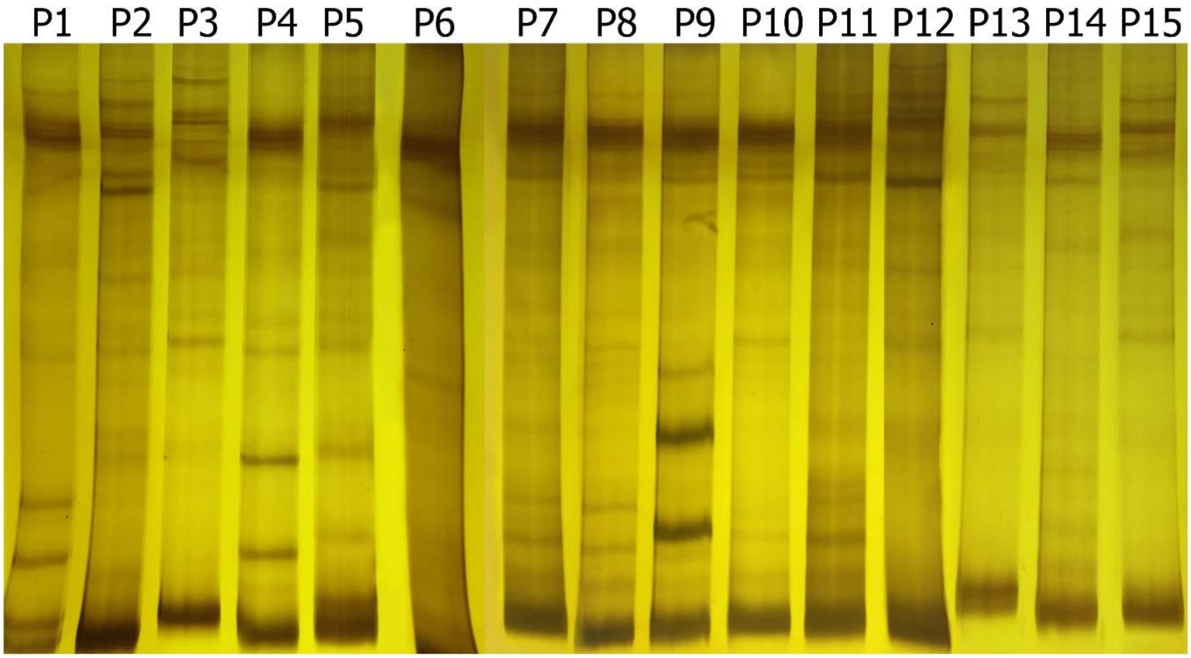

Supplementary Figure S1: Representative single-stranded conformation polymorphism gel showing 15 different profiles (P1–P7).
